# Do Dehydroepiandrosterone (DHEA) and Its Sulfate (DHEAS) Play a Role in the Stress Response in Domestic Animals?

**DOI:** 10.3389/fvets.2020.588835

**Published:** 2020-10-26

**Authors:** Gianfranco Gabai, Paolo Mongillo, Elisa Giaretta, Lieta Marinelli

**Affiliations:** Department of Comparative Biomedicine and Food Science, University of Padua, Legnaro, Italy

**Keywords:** biomarker, comparative endocrinology, DHEA (-S), domestic animal, HPA, reproductive tissues, stress

## Abstract

In animal husbandry, stress is often associated with poor health and welfare. Stress occurs when a physiological control system detects a state of real or presumptive threat to the animal's homeostasis or a failure to control a fitness-critical variable. The definition of stress has mostly relied on glucocorticoids measurement, even though glucocorticoids represent one stress-response system, the hypothalamus-pituitary-adrenocortical axis, which is not precise enough as it is also related to metabolic regulation and activated in non-stressful situations (pleasure, excitement, and arousal). The mammal adrenal can synthesize the androgenic steroid dehydroepiandrosterone (DHEA) and its sulfate metabolite (DHEAS), which have been associated to the stress response in several studies performed mostly in humans and laboratory animals. Although the functions of these steroids are not fully understood, available data suggest their antagonistic effects on glucocorticoids and, in humans, their secretion is affected by stress. This review explores the scientific literature on DHEA and DHEAS release in domestic animals in response to stressors of different nature (inflammatory, physical, or social) and duration, and the extra-adrenal contribution to circulating DHEA. Then, the potential use of DHEA in conjunction with cortisol to improve the definition of the stress phenotype in farmed animals is discussed. Although the focus of this review is on farmed animals, examples from other species are reported when available.

## Introduction

The inability to predict and control the complexity of the environment where an animal lives may result in behavioral, physiological, and immunological changes generating a stress. The stress response is elicited when a real or supposed threat to homeostasis is perceived by the animal, which responds by activating a wide array of mechanisms aiming at restoring homeostasis (1–3). The stress response is an adaptive/physiological response and, indeed, short-term stress responses are mostly positive (1). Conversely, a repeated or continuous activation of the stress response systems for a long time interval can lead to health threatening consequences (1). An unambiguous definition of stress is problematic because many factors can act as stressors and the behavioral and physiological responses to identical environmental conditions may differ among individual animals belonging to the same population (2, 4). In the context of animal husbandry, stress has often a negative connotation and it is associated with poor health or hampered welfare (5, 6).

As the negative effects of stress are associated to chronic or repeated elevation of glucocorticoid hormones in the blood (1), the definition of stressful conditions relies mainly on glucocorticoid measurement also in domestic animals. Nevertheless, glucocorticoid release represents one system which responds to a stressor, the Hypothalamus-Pituitary-Adrenal (HPA) axis. Therefore, analyzing glucocorticoid levels alone gives only a partial picture of animal welfare (6–8). In addition, activation of the HPA axis does not strictly reflect what is termed “stress,” as it is involved in metabolic regulation independent of stressors and physiological arousal is not necessarily linked to negative experiences (1, 6–8). For these reasons, it is unworthy to use the glucocorticoid hormones as the sole stress biomarkers, but they should be measured in conjunction with other physiological and behavioral indicators; rather, why the HPA axis has been activated and the potential physiological consequences of this activation should be considered (1, 8).

The adrenal androgen precursor dehydroepiandrosterone (DHEA) and its sulfate metabolite (DHEAS) are implicated in the short- and long-term stress response, and studies on humans and laboratory animals reported that these steroids show effects that compensate for or oppose those of cortisol (9–12). Therefore, DHEA and DHEAS could also be considered as important players in the stress response and potential biomarkers of stress in domestic animals. However, different animal species can display specific features in the synthesis, regulation and biological role of these steroids to satisfy their own physiological requirements (13).

**Figure 1:**
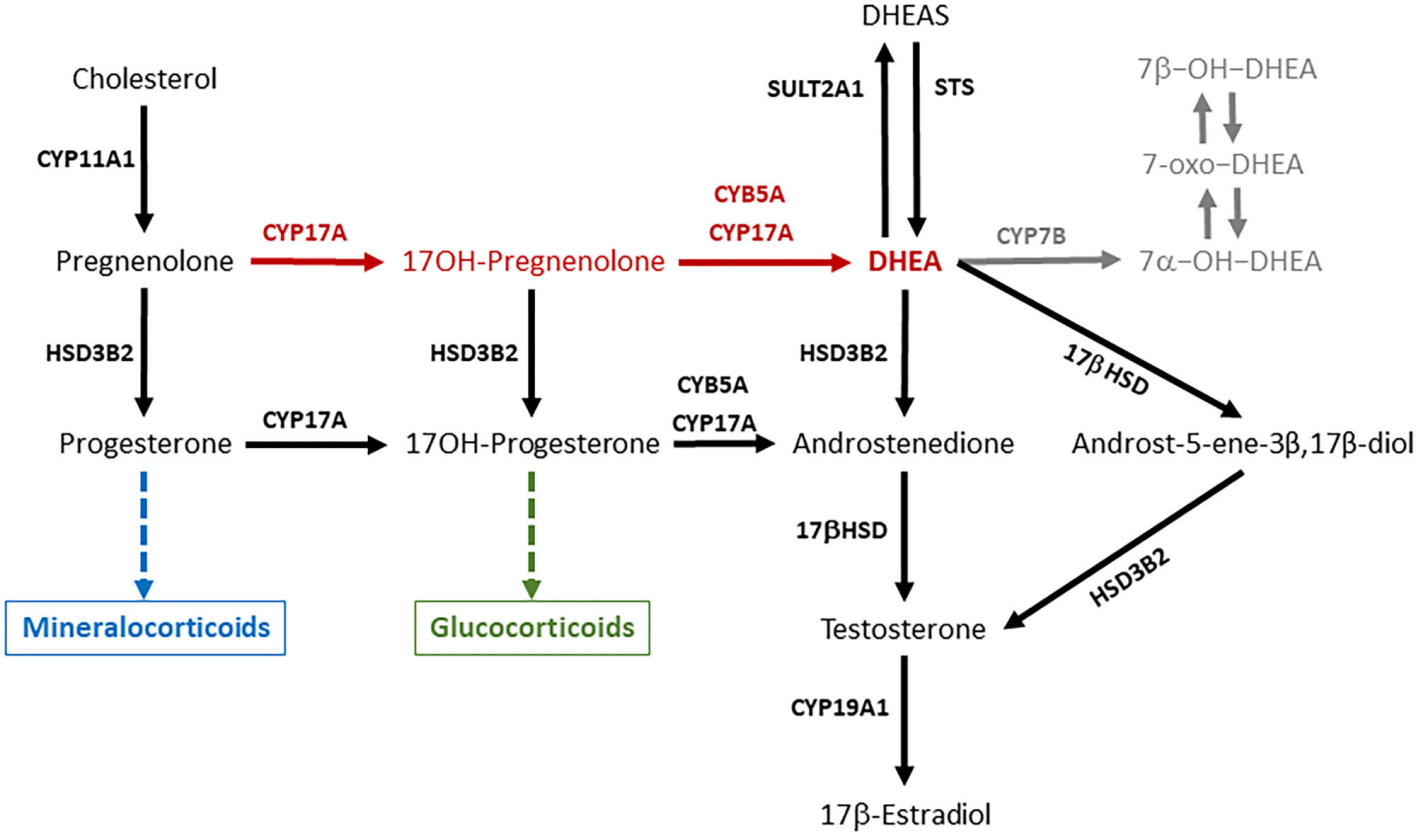
DHEA is synthesized from pregnenolone through the Δ^5^ pathway (red arrows) thanks the activity of the enzyme CYP17A1. DHEA synthesis occurs in two steps, which are both mediated by CYP17A1, which compete for pregnenolone with HSD3B2, the initiator of the Δ^4^ pathway necessary for mineralocorticoid and glucocorticoid production. DHEA can be interconverted into DHEAS and metabolized in several extra-adrenal and extra-gonadal tissues to give rise to biologically active metabolites such as androst-5-ene-3β,17β-diol and oxygenated metabolites (in gray). The ability of a steroidogenic tissue to synthesize DHEA depends on the expression and activity of CYP17A, its interaction with CYB5, and the expression of HSD3B2, which competes with CYP17A for substrates. This figure describes the general pathways involved in DHEA synthesis and metabolism, and pose some emphasis on the main enzymes involved. CYP11A1 is an inner mitochondrial membrane enzyme, also known as cytochrome P450scc (side chain cleavage), which converts cholesterol to pregnenolone. CYP17A, also known as cytochrome P450c17, is the key enzyme of the Δ^5^ pathway that catalyzes the conversion of pregnenolone to DHEA in two steps: (a) 17α-hydroxylation of pregnenolone and progesterone; (b) 17,20-lyase reactions required to break the C17–C20 bond of 17-OH-pregnenolone and 17-OH-progesterone and produce C19-androgens (i.e., DHEA and androstenedione). CYB5A, also known as cytochrome b5, is an allosteric enhancer of the 17,20-lyase activity of CYP17A. HSD3B2, also known as 3β-hydroxysteroid dehydrogenase, Δ^4^/Δ^5^-isomerase (3βHSD) type 2, is the key enzyme of the Δ^4^ pathway, which catalyzes the oxidation of the hydroxyl group on carbon 3 of steroids to a keto group and, simultaneously, the isomerization of the double bond from the B ring (Δ^5^ steroids) to the A ring (Δ^4^ steroids). 17βHSD (17β-hydroxysteroid dehydrogenase) converts androstenedione to testosterone, and DHEA to androst-5-ene-3β,17β-diol. CYP19A1, also known as cytochrome P450aro (aromatase), converts testosterone to 17β-estraidiol and androstenedione to estrone. SULT2A1 (DHEA sulfotransferase) converts DHEA in DHEAS. STS (steroid sulfatase) is the primary enzyme involved in steroid desulfation. CYP7B is the main responsible for the generation of 7α-hydroxylated steroids in diverse extra-hepatic tissues.

DHEA is present in the circulation of primate mammals in large amounts (20–60 nM), while lower amounts (around 1–3 nM or less) of this steroid can be found in the blood of rodents and domestic animals, such as rabbits, dogs, pigs, cattle, sheep, and horses (13, 14). DHEA is synthesized in steroidogenic tissues (adrenals, gonads, and placenta) and in the nervous system from pregnenolone through the Δ^5^ pathway, thanks to the activity of the enzyme cytochrome P450c17 (CYP17A) (Figure 1). This is a microsomal enzyme which has a dual function: it catalyzes both the 17α-hydroxylation of Δ^4^- and Δ^5^-steroids necessary for glucocorticoid production, and the 17,20-lyase reactions required to produce C19-androgens (i.e., DHEA and androstenedione). The 17,20-lyase activity of CYP17A is enhanced by the allosteric regulator cytochrome b5 (CYB5), thus facilitating the conversion of 17α-hydroxypregnenolone to DHEA (15–17).

Most information about DHEA and DHEAS biology in mammals have been obtained from studies performed in humans and laboratory animals, but it is quite clear that differences do exist between primates and rodents. The rat adrenal does not express the CYP17A enzyme and, therefore, does not produce androgen pre-cursors, and corticosterone is the major glucocorticoid in this species (15, 16). The tissue expression of sulfotransferase enzymes responsible for DHEAS production is different between rodents and humans, and care should be taken when translating findings obtained in rodents to human physiology (18). Moreover, differences in the amino acid sequences and function of CYP17A in pig, Guinea pig, and cattle have been reported, which can explain differences observed across species in adrenal androgen synthesis (16). In humans, adrenal androgen secretion is responsive to ACTH and stressors (10). In domestic mammals, adrenal secretion of DHEA in response to ACTH is well-documented “*in vitro*” (19–23). Nevertheless, published “*in vivo*” studies that have explored DHEA and DHEAS release in response to the administration of exogenous ACTH or stressors produced contradictory results [cattle: (6, 24, 25); dog: (26, 27)].

This review explores the scientific literature on DHEA and DHEAS release in domestic animals in response to stressors of different nature (inflammatory, physical, or social) and duration, and the extra-adrenal contribution to circulating DHEA. Then, the potential use of DHEA in conjunction with cortisol to improve the definition of the stress phenotype in farmed animals is discussed. Data obtained from domestic animals are compared to those obtained from rodents, humans and non-human primates. Although the focus of this review is on farmed animals, examples from other species are reported when available.

## Adrenal DHEA/DHEAS Synthesis

The ability of the adrenals to synthesize DHEA varies among species depending on the expression and activity of CYP17A, its interaction with CYB5, and the expression of 3β-hydroxysteroid dehydrogenase type 2 (HSD3B2), which competes with CYP17A for substrates (Figure 1). On one end, the zona reticularis of the primate adrenals has the unique enzyme profile that promotes the efficient DHEA production through the Δ^5^ pathway, and the low expression of HSB3B2 favors flow of substrates toward DHEA (10, 17, 28–30). On the other end, the CYP17A enzyme in the rodent adrenal lacks the 17,20-lyase activity, thus explaining the low DHEA secretion observed in these species (16, 31). In particular in the guinea pig, it was observed that the conversion of 17α-OH-pregnenolone to DHEA is inhibited, while there is a functioning CYB5-responsive 17,20-lyase activity that converts 17α-OH-progesterone to androstenedione (16). In the rabbit (lagomorph), both adrenal 17-hydroxylase and 17,20-lyase activities are lower than those observed in primates (32).

Besides primates and rodents, the adrenals of domestic mammals display different enzyme patterns. The bovine has a C17,20-lyase activity responsive to CYB5 that converts 17α-OH-pregnenolone to DHEA but does not convert 17α-OH-progesterone to androstenedione (16). In addition, the HSD3B2 activity in the bovine adrenal cells is very high and pregnenolone is quickly converted to progesterone with little DHEA formation, even though adrenal cells are exposed to ACTH (33). In the pig the CYP17A has a C17,20-lyase activity responsive to CYB5 that converts 17α-OH-pregnenolone to DHEA and 17α-OH-progesterone to androstenedione (16), and the pig adrenal tissue contains an appreciable amount of DHEA (19). In dogs, the adrenal 17,20-lyase activity is significantly lower as compared to primates (32). However, a decline in plasma DHEA was observed in senior ovariectomized female dogs that probably reflects adrenal DHEA synthesis (34).

In primates, DHEA circulates in the blood mainly in its sulfated form (DHEAS) at micromolar levels. DHEAS is particularly high in humans and chimpanzees (13), and represents a circulating reservoir for DHEA. The conversion of DHEA into DHEAS is primarily catalyzed by the sulfotransferase type 2A1 (SULT2A1) enzyme (Figure 1), although other sulfotransferase enzymes such as SULT1E1 or members of the SULT2B family can catalyze DHEAS synthesis (18). As the zona reticularis of the human and other primate adrenals abundantly expresses SULT2A1 and lacks HSD3B2, it is responsible for the huge DHEAS synthesis (18, 29, 35). Corticotrophin releasing hormone (CRH) and ACTH stimulation enhances SULT2A1 gene expression, and DHEA binding to SULT2A1 protein inhibits enzyme activity (18).

DHEAS is present in the plasma of rat, dog, and pig at nanomolar levels, and it is undetectable (14) or very low (13, 24) in the plasma of ruminants, guinea pig, and rabbit. Moreover, in non-primate mammals the adrenals are not elective sites of DHEA sulfonation. Among domestic mammals, the male pig shows the higher circulating DHEAS concentrations (13). However, SULT2A1 and SULT2B1 are poorly expressed in the boar adrenals in comparison with testes (36), and available data suggest that DHEA sulfonation occurs mainly in testicles and epididymis (36–40). DHEAS is undetectable in plasma of non-pregnant gilts (37) and in the adrenal of adult cycling sows (19). Also the ovarian expression of SULT2A1 and SULT2B1 in the adult cycling female pig is extremely low (36), suggesting that ovaries are poorly involved in steroid sulfonation in the female pig. In the bovine, DHEAS synthesis takes place in the adrenal gland, uterus and placenta, and the bovine placenta seems more active than human placenta in operating steroid sulfonation (41). Considering all the sex and species differences in DHEAS synthesis and origin, researchers should be cautious in using DHEAS as a stress biomarker in non-primate mammals.

## Extra-adrenal DHEA/DHEAS Synthesis

### Gonads

In humans, most of the circulating DHEA and virtually all DHEAS is of adrenal origin, although a portion of circulating DHEA is produced by the gonads in men and women (30). In rodents and domestic mammals, gonads contribute to circulating DHEA but the relative adrenal and gonadal contribution is difficult to extrapolate, such as in the cow (24) and the bitch (34, 42). Moreover, gonadal DHEA synthesis may be regulated by factors independent from those involved in the stress response, and this hypothesis should be carefully considered when analyzing DHEA to study HPA axis activation, as gonadal or placental DHEA production may bias the putative adrenal response.

In the bovine ovary, estrogens are synthesized primarily through the Δ^5^ pathway (Figure 1), and DHEA is the substrate for androstenedione production (43). DHEA and DHEAS concentrations in the peripheral blood of cows do not vary significantly around the time of induced ovulation, and show considerable differences among individual animals (24). Plasma DHEA fluctuations have been observed also in the ovarian veins, which are characterized by inter-subjects variability and no relationship with the day of the estrous cycle (43). Interestingly, DHEA concentrations in the follicular fluid remain unaltered around the LH surge, decrease abruptly 4 h after the LH surge and remain low until ovulation, suggesting an LH dependent inhibition of ovarian DHEA and other androgen synthesis (44). It is worth noting that, in the cow, the CYP17A gene expression is stimulated by cyclic AMP in both the adrenal cortex and ovarian follicle theca, but it is regulated in a tissue-specific fashion (45).

Also in the dog both the ovary and testis give a considerable contribution to circulating DHEA (34, 42) and respond to GnRH agonist administration by reducing DHEA synthesis (42). Indeed, DHEA plasma concentrations are highest in proestrus than in estrus and anestrus in this species (46).

Both DHEA and DHEAS are present in the circulation of the Yucatan mini-pig, a natural breed indigenous of Mexico and closely related to the European wild pig, and DHEAS concentrations are higher in males than females (47). As reported above, the adult male pig produces the highest androgen levels among ungulates, which originates primarily from the testes (38), and pregnenolone is metabolized to DHEA through the Δ^5^ pathway (48). As Leydig cells obtained from adult boar testes respond to human chorionic gonadotropin by increasing DHEA (48) and DHEAS secretion, their concentrations in the blood may increase in response to sex arousal rather than stressors. In the horse, the CYP17A enzyme expression is already detectable in the Leydig cells of pre-pubertal males, and immune-stain intensity increases with age, suggesting that DHAE synthesis occurs in the stallion testis (49). However, an early study found that the 17,20-lyase activity in the Δ^5^ pathway converting 17OH-pregnenolone to DHEA was less active than the 17,20-lyase activity in the Δ^4^ pathway (Figure 1), which converts 17OH-progesterone to androstenedione (50). The low DHEA concentrations observed in the seminal plasma of stallions are in agreement with the greater importance of the Δ^4^ pathway for androgen production in the equine testis (51) and lower contribution of the male gonads to circulating DHEA.

### Placenta

In all species studied so far, the placenta expresses steroidogenic enzymes and it is capable to synthesizing and/or metabolizing steroid hormones. However, important differences among species are present in placental steroidogenesis (52).

The horse placenta expresses low levels of CYP17A (53), and in this respect, it is similar to that of human and some non-human primates (52). The horse fetus produces a considerable amount of DHEA that is probably used by the allantochorion to synthesize estrogens (53). However, a considerable amount of DHEA escapes the placenta, as its concentrations in the maternal circulation are quite high, displaying values around 3–6 nM in early pregnancy, peaking to 50–60 nM in mid-pregnancy, and then decreasing toward the end of pregnancy (54, 55). DHEAS was detected in all mares between 7 and 9 weeks of gestation, when its concentrations were around 27 nM. After the first 3 months of gestation, little or no DHEAS was detectable (56).

The placenta of the rat, pig, sheep, and cow express CYP17A and it is capable of DHEA synthesis (52). The cow placenta is an important source of DHEA, which utilizes mainly the Δ^5^ steroidogenic pathway to produce estrogen (57). In this species, plasma DHEA increases in late pregnancy, probably reflecting the volume and secretory activity of fetal and placental tissues (57), and decreases after parturition (24, 58). However, DHEA concentrations in the maternal plasma are very low in comparison with the mare, and varies between <1 nM in non-pregnant and early pregnant animals, and 4 nM in late pregnant subjects (58).

As DHEA concentrations can be affected by a significant placental contribution, it is of the utmost importance to consider the species and the reproductive status of a female when measuring DHEA in the context of the stress response assessment. This is particularly true when an “accumulation matrix” such as hair is used (6). In cows, for example, the hair DHEA content measured during early post-partum could be affected by the placental DHEA production occurring in late pregnancy (24, 58). This is an important point to investigate before hair DHEA could be used as a stress biomarker, and we are not aware of studies examining such a possibility.

### Nervous System

The capability of the nervous system to synthesize and metabolize steroids is known since the early 1980s, when the observation that DHEAS is present in the male rat brain in a quite large amount led to the discovery of a steroid synthesizing machinery within the nervous system. Brain DHEAS was apparently independent from adrenal secretion, was not affected by long-term ACTH or dexamethasone administration, and by adrenalectomy plus orchiectomy. It was, therefore, proposed that DHEAS is formed or accumulated within the rat brain depending on local mechanisms unrelated to the peripheral steroidogenic tissues (59). Indeed, the term “neurosteroids” was proposed to describe the steroids that accumulates in the nervous system independently from peripheral steroidogenic tissues and can be synthesized “*in situ*,” and this definition applies to DHEA (60).

Although neuro-steroidogenesis seems quite conserved among animal taxa [mammals: (60–66); birds: (67–69)], to the best of our knowledge information about DHEA content and steroidogenic enzyme distribution within the brain of domestic animals is scarce. Little scientific work explored steroid synthesis in the brain of domestic and farmed animals, and most information about neurosteroid synthesis has derived from rodent models. In our opinion, a deeper knowledge of DHEA/DHEAS production and activity within the brain may be important to better understand the adaptation of domesticated animals to husbandry conditions.

In rats the highest levels of DHEA were observed in the spinal cord compared to plasma, hippocampus, cerebral cortex, cerebellum and sciatic nerve, and plasma DHEA levels were not significantly correlated with central nervous system (CNS) tissue levels (70). The pathway of DHEA synthesis in the rodent brain is controversial, as the CYP17A enzyme expression is low in the adult rats and alternative CYP17A-independent pathways were suggested. The presence of hydroxysteroid sulfotransferase activity in the rodent brain suggested that DHEA, which can readily pass the blood–brain barrier, could be locally converted to DHEAS (60, 61). In humans, DHEA and DHEAS can be synthesized “*de novo*” in the CNS, but a significant proportion of steroid metabolites may be also synthesized in the CNS from steroid precursors or directly transported through the blood-brain barrier from the periphery (71).

In several rat, bovine, and human brain model systems, DHEA biosynthesis is mediated by an oxidative stress/Fe^2+^, independent of the CYP17A enzyme. This mechanism is not fully understood and it may be typical of pathologic conditions such as ischemia, trauma, or neurodegeneration, which can generate an oxidative environment and increase oxidative stress (72).

The presence of a neurosteroid sulfatase in the bovine brain (73) suggests that DHEA crossing the blood–brain barrier could be locally converted to DHEAS. Information about steroid metabolizing capabilities of the dog brain is scarce. We are aware that both Purkinje neurons and oligodendrocytes of the dog cerebellar cortex contain steroidogenic enzymes (e.g., 3βHSD) (74). Both DHEA and DHEAS were detected in the cerebrospinal fluid (CSF) of dogs, concentrations of both compounds were higher in males than in females, and DHEAS decreased with increasing age (75).

## Factors Affecting DHEA/DHEAS Concentrations in the Blood

Age is an important factor affecting DHEA and DHEAS secretion in primates. A pre-pubertal increase in circulating DHEA and DHEAS, defined as adrenarche, is observed in humans and some great apes, but not in Old World monkeys; it was hence suggested that adrenarche has evolved to promote brain development (17, 76). In humans, circulating DHEA and DHEAS increase gradually after adrenarche until the mid-twenties and then decline with aging, the adrenals being the main source of these steroids. Interestingly, DHEAS concentrations are higher in males than age-matched females (17).

To the best of our knowledge, data about DHEA and DHEAS concentrations in plasma of aged ungulates are lacking. However, in an “*in vitro*” study it was observed a lower CYP17A expression and a reduced both basal and ACTH-stimulated DHEA release in aged (10–12 year old) bovine adrenal cells (77).

Plasma DHEA concentrations are affected by sex and age also in the dog. In pre-pubertal dogs (<6 month-old), no differences in circulating DHEA could be observed between males and females. In dogs aged between 6 months and 2 years plasma DHEA concentrations were higher in males than females (34). Plasma DHEA increased in post-pubertal compared to pre-pubertal male dogs, even though to a smaller extent than in man. However, plasma DHEA concentrations were almost undetectable in orchiectomized male dogs suggesting a testicular rather than adrenal origin (32). In the study by Mongillo et al. (34), plasma DHEA concentrations were numerically lower in senior (over 8 years-old) male dogs. In females, the age-related reduction in plasma DHEA was statistically significant, confirming the observation of a previous study (46). Interestingly, the contribution of the ovaries to circulating DHEA seems limited, as no differences were observed between intact and ovariectomized females (34). Although this is not within the scope of this review, it is noteworthy that gonadectomy may influence adrenal androgen synthesis, as suggested by observations made in the male rhesus monkey, where adrenal androgen secretion was greater in castrates than intact males (76). This issue has not been investigated in domestic mammals so far, and it would merit great attention, as it may imply the existence of compensatory mechanisms that could have an impact on animal adaptation.

In humans, DHEA displays diurnal patterns of secretion, in some circumstances resembling those of testosterone and cortisol (78). Similarly to cortisol, DHEA shows the highest concentrations in the morning and the lowest in the evening (10, 78), although DHEA's diurnal rhythm is less marked and lacks the surge observed in plasma cortisol after awakening (10, 79). Differently from DHEA and cortisol, circulating DHEAS does not display visible diurnal rhythm nor day-to-day variation (10). In healthy subjects, DHEA release is episodic and apparently synchronous with cortisol release, while DHEAS release shows less pronounced fluctuations (80, 81). Aging seems to have a strong impact not only in DHEA concentrations, but also in DHEA pattern of secretion, that tends to disappear in older subjects (78). Although diurnal patterns of DHEA and DHEAS may be affected by chronic stress, nutritional behaviors, physical exercise, drugs and sleep deprivation or shift, psychiatric and neurologic diseases, cancer and other complex pathologies, conclusive observations about the effects of these factors are not possible due to the lack of studies performed in humans (78), and to the best of our knowledge, in animals.

We analyzed the episodic release of cortisol, DHEA and DHEAS in six lactating, non-pregnant cows in their 3rd month of lactation over a period of 8 h between 10:00 and 18:00 h (24). Secretion of both DHEA and DHEAS was episodic, although mean plasma DHEA concentrations and pulse amplitude were significantly higher than those of DHEAS. Relationship between neither DHEA nor DHEAS and cortisol were observed, suggesting that cortisol and DHEA/DHEAS release are desynchronized. This study was not suitable to detect any circadian pattern of DHEA and DHEAS release. We are not aware of other studies investigating the daily patterns of DHEA and DHEAS release in domestic animals. In our opinion, the understanding of the DHEA role in the stress response necessarily implies a deeper knowledge of its secretion patterns in blood and saliva, which is increasingly used as biological matrix to study short-term steroid hormone secretion (6, 78, 82).

## DHEA/DHEAS Release in Response to ACTH and Stressors

In humans, circulating DHEA increases in response to ACTH (10, 30), and the sensitivity of DHEA secretion to ACTH is similar to cortisol (30, 83). In addition, the magnitude of the DHEA response to ACTH increases with age (84). In rodents, DHEA release is stimulated by ACTH. Mature female rat adrenal cells produce DHEA in response to ACTH and the response was enhanced when a crude extract from rat pituitary gland was added in culture. These observations suggested the existence of a pituitary adrenal androgen stimulating factor (85). In male and female rats, CRH and ACTH administration stimulates DHEA increase in both plasma and brain (86). DHEA increased also in plasma and adrenal tissue in Mongolian gerbils (*Meriones unguiculatus*) in response to confinement stress (87).

Studies performed “*in vitro*” suggested that androgen release by the bovine adrenal is increased by ACTH (20–23) (Table 1). In some of those studies (21–23), however, androgen concentrations were measured by a commercial adrenal androgen radioimmunoassay that employed an antibody that reacts with all adrenal androgens (DHEA, DHEAS, and androstenedione). Therefore, it is not easy to work out the contribution of DHEA and DHEAS to the androgen pool (20–23). Interestingly, ACTH differently affects steroidogenic enzymes in the human and bovine adrenals. In particular, 3βHSD activity after induction with ACTH is higher in the bovine than in human; as a consequence, bovine adrenal cells convert pregnenolone to progesterone, which can be converted to 17-hydroxyprogesterone, with minimal formation of DHEA (33).

**Table 1:** Hormones and cytokines involved in the regulation of adrenal DHEA/DHEAS synthesis.

**Factor**	**Species**		**Source**	**End-point**	**Effect(s)**	**References**
ACTH	Human	Review		DHEA secretion	Stimulation	(10, 30)
	Human	*In vivo*	Healthy male and female	Blood DHEA	Stimulation	(83)
	Rat	*In vivo*	Acute administration	Blood and brain DHEA	Stimulation	(86)
	Rat	*In vitro*	Adrenal cells	DHEA in culture medium	Stimulation	(85)
	Bovine	*In vitro*	Adrenal cells	Androgens(*) in culture medium	Stimulation	(21–23)
	Bovine	*In vitro*	Adrenal cells	Steroidogenic enzyme activities	Increase 3βHSD activity, increase adrenal DHEA utilization	(33)
	Bovine	*In vitro*	Adrenal cells	DHEA in culture medium	Stimulation	(77, 88)
	Bovine	*In vivo*	Acute administration	Blood DHEA	Irresponsive	(24, 25)
	Bovine	*In vivo*	Chronic administration	Blood DHEA	Increased mean concentrations and pulse amplitude	(24)
	Dog	*In vivo*	Acute administration	Blood DHEAS	Stimulation	(26)
	Bird (Cardinal)	*In vivo*	Acute administration	Blood DHEA	Irresponsive	(89)
PRL	Baboon	*In vivo*	Acute (90 min infusion)	Blood DHEA and DHEAS	Stimulation	(90)
	Bovine	*In vitro*	Adrenal cells	DHEA and DHEAS in culture medium	Synergistic to ACTH—stimulation	(91)
	Swine	*In vivo*	Chronic administration	DHEA in adrenal tissue	Irresponsive	(19)
LIF	Bovine	*In vitro*	Adrenal cells	Androgens(*) in culture medium	Inhibition (not stimulated cells) Inhibition (ACTH-stimulated cells)	(22)
IL4	Bovine	*In vitro*	Adrenal cells	Androgens(*) in culture medium	Irresponsive (not stimulated cells) Inhibition (ACTH-stimulated cells)	(21)
IL6	Bovine	*In vitro*	Adrenal cells	Androgens(*) in culture medium	Inhibition (not stimulated cells) Inhibition (ACTH-stimulated cells)	(23)
	Bovine	Review	Adrenal cells	DHEA in culture medium	Inhibition (not stimulated cells) Inhibition (ACTH-stimulated cells)	(92)
	Human	Review	Adrenal cells	DHEA in culture medium	Stimulation	(92)
TNFα	Human	Review	Fetal adrenal cells	DHEA in culture medium	Modest inhibition	(92)
	Bovine	Review	Adrenal cells	DHEA in culture medium	Inhibition (not stimulated cells) Inhibition (ACTH-stimulated cells)	(92)
SFA	Bovine	*In vitro*	Adrenal cells	DHEA in culture medium	Synergistic to ACTH—stimulation	(88)

(*) Androgens: total androgens (DHEA, DHEAS, androstenedione) measured in the culture medium. ACTH, adrenocorticotropic hormone; IL4, Interleukin 4; IL6, Interleukin 6; LIF, Leukemia Inhibitory Factor; PRL, prolactin; SFA, saturated Fatty Acids; TNFα, Tumor Necrosis Factor alpha; 3βHSD, 3β-hydroxysteroid dehydrogenase, Δ^4^/Δ^5^-isomerase enzyme.

The release of DHEA in response to ACTH “*in vivo*” produced contradictory results in some species. In dairy cows, Jurkovich et al. (25) observed that DHEA was irresponsive to ACTH treatment. Moreover, Marinelli et al. (24) observed that a single dose of an ACTH agonist did not induce an acute increase in both circulating DHEA and DHEAS. The same authors (24) observed that mean DHEA plasma concentrations and peak amplitude were higher in animals treated with the same ACTH agonist every 12 h for 6 days. In dogs, an increase in circulating DHEAS was observed in neutered, but not in intact, males 1 h after the administration of an ACTH analog (26). In this paper, DHEAS but not DHEA was measured, and DHEAS secretion may respond slower to ACTH. Moreover, gonadal DHEAS contribution may have masked the effect of ACTH in intact males. Administration of exogenous ACTH failed to alter DHEA levels also in birds (e.g., *Cardinalis cardinalis*) (89).

These observations support the hypothesis that ACTH is not a primary secretagogue for DHEA, and that other factors may have a significant role in the regulation of DHEA and DHEAS secretion (23, 24, 89). It is possible that adrenal DHEA synthesis requires the presence of ACTH, but the basal ACTH-dependent output could be either enhanced or inhibited by other factors. As an example, ACTH activity in bovine adrenocortical cells can be regulated by the interaction with growth factors like IGF-1 (enhancing effect) and TGFβ (inhibiting effect), which can influence the expression of ACTH receptor, CYP17A, 3βHSD, and steroidogenic acute regulatory protein (StAR), the latter being the most affected (20). Moreover, overexposure to saturated fatty acids increases DHEA production by bovine fasciculate/reticularis cells in culture stimulated with ACTH (88). Also prolactin, when administered in combination with ACTH to a monolayer culture of bovine adrenal cells, enhanced the ACTH-dependent DHEAS and DHEA, but not androstenedione, secretion (91). Interestingly, the effects of prolactin on adrenal steroidogenesis seems species dependent, as prolactin administration can alter adrenal cortisol, but not DHEA, secretion in the pig (19), while increased DHEA/DHEAS, but not cortisol, were found in hyperprolactinemic women (90), and prolactin administration to infant baboons specifically increased adrenal androgen production (93).

The hypothesis that ACTH is not a primary secretagogue for DHEA becomes even stronger when examining DHEA/DHEAS release in response to different kinds of stress. In humans, changes in DHEA and DHEAS concentrations associated with several disease states and immunological factors, diet and metabolic function, stress, CNS functions and psychiatric disorders have been extensively reviewed (10, 78, 94, 95). The general lesson learnt from the human is that acute and chronic stressful situations or diseases can differently affect plasma DHEA and DHEAS concentrations. A decrease in both steroids can be observed in chronic inflammation, and cytokines such as TNFα and IL6 may play a regulatory role (96). Similarly, increased circulating DHEA and DHEAS levels can be observed in response to an acute psychosocial stressor (97). Chronic exposure to prolonged psychosocial stress does not affect blood DHEA concentrations, but it seems that DHEAS production in response to a subsequent acute stress is blunted (98).

The stress response is a complex phenomenon. Several neuronal circuits from different brain districts (hippocampus, amygdala, and the pre-frontal cortex) are implicated in the regulation of CRH and arginine vasopressin (AVP), which in turn stimulate ACTH and glucocorticoid release. Glucocorticoids regulate their own secretion by a feedback inhibition mechanism (6). However, the stress response is not merely related to HPA axis activation, and other factors help the animals to adapt to stressors.

### The Role of the Immune System

The nervous, endocrine, and immune systems cooperate in elaborating the appropriate behavioral and physiological responses to environmental challenges (99) and, among these, the immune system plays a prominent role in regulating the responses to acute and chronic stressors (5, 100).

It is well-known that cytokines interfere with steroidogenesis in a systemic and complex manner. Studies performed mainly in laboratory animals [extensively reviewed in (100)] indicate that IL1 and other cytokines such as leukemia inhibitory factor (LIF), IL6 and TNFα can influence ACTH, and glucocorticoid hormones release by acting at the brain or pituitary levels (92, 100). The sensitivity of the HPA axis to cytokines, IL1 in particular, has been assessed in birds (chicken), rodents, sheep, and primates, suggesting that this adaptive response is highly conserved among animal taxa (100). In addition, cytokines can influence development, function, and hormone production of the adrenals, testes, and ovaries (101). In the context of this review, the effects of several cytokines have been studied in bovine adrenocortical cells in a series of “*in vitro*” experiments. In few cases, they were compared with the effects observed in humans and rodents (Table 1).

The adrenals of rat, bovine, and humans express both IL6 and TNFα and their receptors, even though the topography of their expression is not consistent among species (92). An autocrine/paracrine regulation of the adrenal steroidogenesis can be observed, although IL6 and TNFα induce different effects on DHEA release in the human and bovine adrenals.

Interleukin-6 increases DHEA release from human, but decreases DHEA secretion from bovine adrenal cells (92). The effects of IL6 in the bovine adrenals are quite complex, as this cytokine inhibits basal and ACTH-stimulated androgen release from the zona reticularis in a dose and time dependent manner; while increased basal and ACTH-stimulated androgen release from mixed adrenocortical cells. In the zona reticularis, IL6 decreased the expression of StAR protein, cholesterol side chain cleavage (CYP11A1) enzyme, CYP17A, HSD3B2, nuclear factor steroidogenic factor 1 (SF-1), that stimulates steroidogenesis, and enhanced the expression of DAX-1, a nuclear factor that inhibits steroidogenesis (23).

Tumor Necrosis Factor-α does not affect DHEA secretion from fetal human adrenocortical cells, but inhibits basal and ACTH-stimulated DHEA release from adult bovine adrenal cell. This difference in TNFα action on DHEA secretion observed between human and bovine may be related to the stage of cell differentiation (human fetal vs. bovine adult adrenocortical cells) or a real species difference.

Interleukin-4 (IL4), IL4 receptor (IL4R), LIF and LIF receptor (LIFR) are also expressed in the bovine adrenal cortex. Both cytokines negatively affected ACTH-stimulated androgen release in a dose and time dependent manner from bovine zona reticularis cells. LIF also inhibited basal adrenal androgen release (21, 22).

### Immuno-Stressors in Domestic Animals “*in vivo*”

In domestic animals, the scientific literature about DHEA release in response to stressful situations is quite scarce. DHEA release has been explored, perhaps not systematically, in response to uterine infections, lameness, infectious diseases, transportation and other potentially stressful situations.

In clinically healthy post-partum mares, DHEA concentrations were higher in animals with lower degree of uterine involution (102). Perhaps, the delay in uterine involution was accompanied by a mild oxinflammatory condition (103, 104) as revealed by slightly higher plasma serum amyloid A (SAA) concentration and oxidative stress indicators. Blood DHEA concentrations were higher also in cows with metritis, and DHEA concentrations were even higher in cows with endometritis and leukopenia. The latter displayed also lower cortisol/DHEA ratio than healthy cows. Authors concluded that DHEA and the cortisol/DHEA ratio could represent an anti-inflammatory signal during prolonged inflammation and a putative prognostic biomarker for evaluating disease severity (105). It is worth noting that inflammatory events can have long term effects on ovarian steroidogenesis in cattle. As an example, in cows suffering of subclinical endometritis in early post-partum DHEA concentrations were decreased in the follicular fluid of the largest follicles around 2 months after calving (106).

In cows, lameness can have either infectious (e.g., foot rot, digital dermatitis, and interdigital dermatitis) or non-infectious (e.g., sole ulcers and white line disease) etiology (107), it is painful for the animals and it is considered as a cause of chronic stress (108). Few papers investigated the HPA response and DHEA release in lame dairy cows (25, 109–111).

In one study (109), authors observed a decrease in serum DHEA and higher cortisol/DHEA ratio in lame compared to healthy cows. Lame animals were selected based on abnormal posture and the presence of visible lesions on at least one limb, but animals with different causes of lameness (sole ulcers, footrot, sole bruising, and interdigital dermatitis) were included. Therefore, it is likely that pain was the common characteristic among lame animals, as they displayed sickness behaviors, and spent less time eating and ruminating, and more time performing self-grooming. Conversely, an increase in both serum cortisol and DHEA was observed in lame cows due to presence of sole ulcers only and displaying a higher neutrophil percentage and a numerically lower lymphocyte percentage suggestive of inflammatory chronic stress (110). Interestingly, the cortisol/DHEA ratio was numerically higher in lame cows also in this study. However, the same group did not observe any differences in DHEA and cortisol, neither in cortisol/DHEA ratio, between healthy animals and cows with sole hemorrhages (111). Authors explained the lack of DHEA and cortisol response by the multiple factors causing sole hemorrhages, as pain might be the only common feature among cows suffering of this disease. Also Jurkovich et al. (25) observed that DHEA concentrations did not differ between lame and not lame cows. The main limitation of this study resides in the way that groups were formed. Animals were allocated into five groups, from non-lame to severely lame cows, based on Locomotion scoring (112), and without considering the different causes of lameness, such as sole ulcer, toe necrosis, white line abscess, and inter digital phlegmon.

Those studies, although all of them examined DHEA release in lame animals, are far from being conclusive, and are quite difficult to compare. The etiology of lameness and experimental designs were heterogeneous and, in the light of the potential effects of the immune system on DHEA release, the inflammatory conditions of the animals involved in the studies has been poorly characterized. Few information about the duration of the disease, pain and inflammatory conditions have been reported, and animals with chronic and acute inflammation may have been allocated within the same experimental groups. In three out four papers, serum haptoglobin was measured to assess the inflammatory status. Haptoglobin is a major acute phase protein in the bovine, and in healthy cattle its serum concentration is <0.02 mg/ml and it can increase to more than 2 mg/ml within 2 days of infection (113). It is worth noting that serum haptoglobin in the three studies were always higher than 1 mg/ml, and quite similar between lame and not-lame animals (25, 110, 111). In one study (111), a difference in serum haptoglobin was found between animals in category 1 (encompassing animals having sole hemorrhage score of 1 and 2) and in category 3 (having a maximum score of 4 or 5). In the same study, authors examined the expression of a panel of cytokines in peripheral lymphocytes, but did not find any difference between mild and severe sole hemorrhage. This study was characterized by a low number of animals, which did not allow a proper categorization. Finally, in one paper (110) different expression levels of IL1α, IL1β, and IL10 were found in peripheral lymphocytes between lame and not-lame cows; conversely, Almeida et al. (109) did not observe any difference in the expression of IL1β in peripheral lymphocytes. In future studies, the presence of multiple etiologies and the characterization of unknown chronic inflammation due to lameness should be carefully considered, and a larger number of homogeneous subjects should be enrolled. However, these studies suggest that DHEA and cortisol measurements, in combination with the observation of locomotion and sickness behaviors, might help targeting animals needing pain relief and facilitate the monitoring of lameness treatments.

Circulating DHEAS is higher in both male and female dogs infected with *Ehrlichia canis* compared with healthy counterparts (114). Conversely, plasma concentrations of DHEA are lower in cats infected by feline leukemia virus (FeLV) or feline immunodeficiency virus (FIV), and animals infected by both retroviruses had significantly lower DHEA plasma values than monoinfected cats (115). Unfortunately, in these experiments authors did not measure circulating cytokines. Tejerizo et al. (115) hypothesized an indirect cytokine-mediated effect on steroidogenesis, as they reported that both FeLV and FIV infections stimulate the release of several proinflammatory cytokines, such as IL-1, IL-6 and TNFα, which may affect adrenal and gonadal steroidogenesis (101). Authors suggested that the regulation in steroid secretion observed in FeLV and FIV infected cats resembles what can be observed in HIV infected humans (116).

Plasma DHEA concentrations were higher in female dogs with inflammatory mammary carcinomas (IMC) and non-IMC compared with healthy subjects, and in IMC compared with non-IMC dogs. Moreover, serum DHEA was highly correlated with DHEA content in tumor tissue (117). These observations suggest that tumor tissue can produce DHEA and other androgens, and that the steroidogenic potential is higher in IMC. Interestingly, the same research group observed an increase in concentrations of IL8 in serum, and IL10 in serum and tumor tissue in dogs with IMC compared with non-IMC animals (118). IL8, in particular, is one of key inflammation regulatory proteins in neutrophils, and it is involved in ovarian follicle development and steroidogenesis (101). These observations support the hypothesis that cytokines, IL8 in particular, affect DHEA (and generally androgen) synthesis also in extra-adrenal steroidogenic tissues.

In foals affected by neonatal maladjustment syndrome (NMS, characterized by neuronal failure resulting from hypoxia and ischemia of the brain that occurs shortly before, during or after parturition), plasma DHEA concentrations were higher at 24 and 48 h of age, while in foals with other neonatal diseases plasma DHEA concentrations were higher at 24 h only (119).

### Physical Stressors

Modifications in blood DHEA concentrations have been observed also in animals exposed to physical stressors, not apparently linked to immune system modifications. However, it is worth remembering that cytokine release is not confined to inflammation, injury or infection, but their release can be altered also during physical and psychological stress (100). Therefore, an effect of cytokines on DHEA/DHEAS release cannot be aprioristically excluded even in the case of physical and psychological stressors. As an example, a relationship among HPA axis activation, DHEA release and immune response can be observed in bulls exposed to transportation stress, where cortisol concentrations peaked and DHEA concentration reached nadir after 4.5 h of transportation. At the same time, neutrophil count increased and genes involved in neutrophil adhesion, chemotaxis and activation, such as IL8, were upregulated (120).

Blood DHEA, but not DHEAS, increased in pre-pubertal gilts underwent surgical procedures for hearth catheterization, in particular following pre-medication, intubation, and induction of anesthesia (27). Authors attributed to surgical stress the increase in circulating DHEA; however, available information does not allow any speculation about putative effect of the immune system or other factors. Plasma DHEA levels were studied in a porcine two-stage model of trauma and delayed sepsis, where anesthetized, ventilated pigs were subjected to local hind-limb trauma and hemorrhage, were resuscitated after 1 h and received *Escherichia coli* LPS after 75 h. DHEA concentrations increased 24 h after trauma, but became lower than basal concentrations 1 h after LPS administration (121).

Stressors could affect DHEA secretion also by indirect endocrine mechanisms. As an example, in dairy cows overstocking for 2 weeks during the pre-partum period significantly increased plasma DHEA concentrations, preceded by an increase in plasma cortisol (122). It is possible that the increase in glucocorticoids due to overstocking stimulated the placental CYP17A enzyme (123, 124) leading to a more efficient conversion of pregnenolone into DHEA. Conversely, alterations in both DHEA and cortisol were not observed in beef cows following abrupt weaning and subsequent housing, despite modifications in the neutrophil number, interferon-γ and haptoglobin secretion suggested an acute stress response in cows post-weaning (125). These observations support the hypothesis that the duration of the stressor can be an important factor affecting hormone interactions and, thus, DHEA concentrations.

## A Biological Role for DHEA and DHEAS in Domestic Mammals?

Although an extensive review of the biological actions of DHEA/DHEAS is beyond the scope of this paper, we would like to offer few tips to stimulate the research on this field in species different from the human and laboratory rodents.

One prominent function of DHAE and DHEAS is their role as prohormones used by several non-endocrine peripheral tissues for the synthesis of sex hormones (androgens and estrogens). Transformation of DHEA into androgens and/or estrogens occurs in peripheral target tissues and depends upon the levels of expression of steroidogenic and metabolizing enzymes in those tissues. This high secretion rate of sex steroids from adrenal DHEA is typical of human and non-human primates, and it is different from other animal models (e.g., rats, mice, and guinea pigs), where sex steroid secretion occurs mostly in the gonads (126, 127).

Besides their role as prohormones, DHEA and DHEAS are believed to possess a wide range of biological actions in different biological systems. In humans, DHEA displays putative pleiotropic effects: it modulates endothelial function, reduces inflammation, improves insulin sensitivity, blood flow, cellular immunity, body composition, bone metabolism, sexual function, and physical strength, and provides neuroprotection, improves cognitive function, and memory enhancement. These functions have been extensively reviewed elsewhere (11, 128–131), and much information has been obtained from studies about DHEA supplementation in humans and laboratory animals (130).

DHEA/DHEAS have putative beneficial effects on heart and vascular system (128), which seems at least in part conserved among mammals. Indeed, effects of DHEA in the regulation the vascular system have been observed in the pig, where the intravenous infusion of DHEA causes coronary (132), and mesenteric, renal and iliac vasoconstriction (133) through the inhibition of a vasodilatory ß-adrenergic receptor-mediated effect, possibly related to the release of nitric oxide (132, 133). Moreover, DHEA can affect proliferation of bovine aortic endothelial cells in a dose-dependent manner, through a mechanism involving the synthesis of hydrogen peroxide (134).

DHEA may also have a role in the regulation of intermediary metabolism (anti-obesity and anti-diabetogenic actions) (128), which has been observed in the Yucatan mini pig, where the administration of DHEA resulted in an increased energy expenditure and lipid utilization. In addition, DHEA administration reduced fatty acid release in response to epinephrine, but did not affect the outcome of glucose-tolerance test (47). Anti-obesity properties of DHEA have been tested also in the dog. The administration of DHEA in combination with a low-fat/high-fiber (caloric restricted) diet in spontaneously obese, euthyroid dogs resulted in a faster rate of weight loss than in caloric restricted diet alone. In addition, DHEA showed hypocholesterolemic activity, particularly affecting the LDL cholesterol, and a decrease in serum thyroid hormones was observed in dogs receiving DHEA (135).

DHEA has a role in the regulation of the several immune system functions, which has been extensively reviewed in (9, 131). DHEA supplementation to laboratory rodents showed positive effects on disease parameters and organism survival, supporting the hypothesis of the anti-inflammatory effects of this steroid. Indeed, DHEA may be involved in the downregulation of the complement cascade; it can affect cytokine production, downregulating inflammatory cytokines and upregulating the anti-inflammatory IL-2 synthesis, thus opposing cortisol effects; it can enhance lymphocyte proliferation, and increase T cell and NK cell cytotoxicity (131). However, it is important to bear in mind that many results derives from “*in vitro*” experiments that may not reflect DHEA concentrations and physiological relationships among tissues and chemical messengers “*in vivo*.” For this reason, a clear picture of DHEA interactions with the immune system is difficult to draw, as DHEA interacts with other hormones and clear independent mechanisms of action have not been described so far (131).

Positive effects of DHEA/DHEAS on the immune response in domestic animals have been observed in few published articles. The action of 1,25-dihydroxyvitamin D(3) and DHEA administered alone or in combination was examined in pigs immunized with human serum albumin. DHEA decreased the IgM serum response and enhanced the IgG2 and IgG serum responses (136). In another study, DHEAS supplementation increased the responsiveness of young pigs to antigenic challenge, counteracted the negative effects of dexamethasone, resulting beneficial for their immune function (137). Immunoprotective and neuroprotective actions of DHEA were observed also in aged dogs, where animals treated daily for 7 months with DHEA had reduced DNA damage in the brain and peripheral blood lymphocytes (138). In cats infected with the FIV, DHEAS treatment reduced inflammatory gene transcripts (IL1β, TNFα, CD3ε, and GFAP) in brain, increased CD4(+) T-cell levels and prevented neurobehavioral deficits and neuronal loss (139).

Since the recognition of the steroid synthetizing capability within the nervous system, a great deal of scientific literature explored the putative actions of DHEA/DHEAS in the nervous system, where these compounds can display a plethora of biological activities and, for this reason, they were defined as neuroactive neurosteroids. DHEA and DHEAS interact with several major receptor systems in the brain (e.g., sigma, glutamate, and GABA-A receptors), and it is hypothesized that they are involved in many important brain functions, such as neuronal plasticity and survival, neuroinflammation, cognition and regulation of some behaviors. Moreover, many scientific works suggested potential preventive and therapeutic actions in different neuropsychiatric and neurodegenerative disorders (60, 61, 63, 65, 66).

Studies in diverse avian and mammalian species suggest that DHEA is important for behavior regulation. For example, DHEA could be responsible for the expression of aggressive behavior when gonadal testosterone synthesis is low, as it can be converted into active sex steroids within the brain (62).

Based on observations performed in rodents, some metabolites of DHEA (Figure 1) may carry out most of the functions traditionally attributed to DHEA (11, 131), and the capability of metabolizing DHEA was found also in peripheral tissues of domestic species. Minced mammary tissues from non-lactating/non-pregnant, non-lactating/pregnant, mid and late-lactating dairy cows showed a certain steroidogenic capability, as tissue incubation with [14C]-labeled DHEA gave origin of several androgens, 5-androstene-3β,17β-diol, androstenedione, and testosterone among them (140). The same androgens could be detected following the incubation of DHEA with bovine liver slices (141). Moreover, the swine liver can metabolize DHEA into several active compounds (7α-OH-DHEA, 7β-OH-DHEA, and 7-oxo-DHEA) (142). The same metabolites are produced in the liver, intestine and brain of rats, mice, and humans (11). In general, 7-oxygenated steroids are widespread in mammals, birds, and fish (143). DHEA and 5-androstene-3β,17β-diol suppress inflammation similarly to hydrocortisone, but increase the levels of the TH1 cytokines (e.g., IL2, IL3, and IFNγ), and help maintaining the TH1/TH2 balance and immune homeostasis. Therefore, these steroids up-regulate immune resistance and protect the host from lethal infection by RNA and DNA viruses, Gram positive and Gram negative bacteria, parasitic infections, and stress mediated immune suppression (9).

## Conclusions

Despite the extensive scientific literature about DHEA and DHEAS, their role has not fully elucidated, and a wide range of activities has been attributed to these steroids. DHEA and DHEAS synthesis and release are peculiar in primate mammals, even though several regulatory factors seem quite conserved among animal taxa. Nevertheless, similar does not mean equal, and the biological properties observed in one animal taxa cannot be straightforward translated to another. A systematic comparative investigation on the biology of these steroids is lacking. Often, scientific works exploring DHEA and/or DHEAS release in domestic mammals did not carefully consider factors that could affect this phenomenon, such as inflammatory and reproductive status, and the time-interval (chronicity) of exposure to stressors.

Studies in humans suggest that DHEA may better reflect the response to acute stress, while DHEAS variations are associated to only long-term perceived stress (10). However, actions of DHEA in the animal stress response is presumably different from that in humans, and data obtained in animals and reviewed in this paper do not support the hypothesis that DHEAS is a good stress indicators, at least in ungulates. In any case, a deeper knowledge on patterns of secretion of DHEA in domestic animals would provide necessary information for sounder conclusions on the description of the stress phenotype.

The most intriguing aspect of DHEA and DHEAS is their antagonistic actions to glucocorticoids. However, this research requires a deeper understanding of the co-actions of these hormones on physiological systems (9). It is believed that the phenotypic response to a stressor could be better described by expressing both steroids simultaneously as glucocorticoid/DHEA ratio, which may be an important indicator of the whole animal response (10). However, one should interpret with care hormone ratios, which should be analyzed with a proper statistical approach (144).

Finally, a better definition of the stress phenotype will be achieved by studying the downstream responses of both glucocorticoids and DHEA (6). In farmed animals, studies performed in the context of the war against illegal hormone or pro-hormone treatments may give additional information about DHEA release (145), metabolism (141) and action (146), providing that studies would be extended to tissues other than the liver.

## Author Contributions

All authors listed have made a substantial, direct and intellectual contribution to the work, and approved it for publication.

## Conflict of Interest

The authors declare that the research was conducted in the absence of any commercial or financial relationships that could be construed as a potential conflict of interest.

## References

[1] Ralph CR Tilbrook AJ Invited review: the usefulness of measuring glucocorticoids for assessing animal welfare. J Anim Sci. (2016) 94:457–70. 10.2527/jas.2015-9645 27065116

[2] Colditz IG. Objecthood, agency and mutualism in valenced farm animal environments. Animals. (2018) 8:50. 10.3390/ani8040050 29614016 PMC5946134

[3] Del Giudice M Buck CL Chaby LE Gormally BM Taff CC Thawley CJ. What is stress? A systems perspective. Integr Comp Biol. (2018) 58:1019–32. 10.1093/icb/icy114 30204874

[4] Romero LM Platts SH Schoech SJ Wada H Crespi E Martin LB Buck CL. Understanding stress in the healthy animal-potential paths for progress. Stress. (2015) 18:491–7. 10.3109/10253890.2015.1073255 26365223

[5] Rohleder N. Stress and inflammation—the need to address the gap in the transition between acute and chronic stress effects. Psychoneuroendocrinology. (2019) 105:164–71. 10.1016/j.psyneuen.2019.02.021 30826163

[6] De Almeida AM Zachut M Hernández-Castellano LE Šperanda M Gabai G Mobasheri A. Biomarkers of fitness and welfare in dairy animals: healthy living. J Dairy Res. (2019) 86:379–87. 10.1017/S0022029919000803 31744555

[7] Mormède P Andanson S Aupérin B Beerda B Guémené D Malmkvist J. Exploration of the hypothalamic-pituitary-adrenal function as a tool to evaluate animal welfare. Physiol Behav. (2007) 92:317–39. 10.1016/j.physbeh.2006.12.003 17234221

[8] Otovic P Hutchinson E. Limits to using HPA axis activity as an indication of animal welfare. Altex. (2015) 32:41–50. 10.14573/altex.1406161 25418851

[9] Loria RM Ben-Nathan D. Protective effects of DHEA and AED against viral, bacterial and parasitic infections. Isr J Vet Med. (2011) 66:119–29. Available online at: http://www.ijvm.org.il/sites/default/files/protective_effects_of_dhea_and_aed_december_book_en1-3.pdf9264155

[10] Kamin HS Kertes DA. Cortisol and DHEA in development and psychopathology. Horm Behav. (2017) 89:69–85. 10.1016/j.yhbeh.2016.11.018 27979632

[11] El Kihel L. Oxidative metabolism of dehydroepiandrosterone (DHEA) and biologically active oxygenated metabolites of DHEA and epiandrosterone (EpiA)—recent reports. Steroids. (2012) 77:10–26. 10.1016/j.steroids.2011.09.008 22037250

[12] Sahu P Gidwani B Dhongade HJ. Pharmacological activities of dehydroepiandrosterone: a review. Steroids. (2020) 153:108507. 10.1016/j.steroids.2019.108507 31586606

[13] Rege J Garber S Conley AJ Elsey RM Turcu AF Auchus RJ Rainey WE. Circulating 11-oxygenated androgens across species. J Steroid Biochem Mol Biol. (2019) 190:242–9. 10.1016/j.jsbmb.2019.04.005 30959151 PMC6733521

[14] Feher T Bodrogi L Feher KG Poteczin E Kolcsey IS. Free and solvolysable dehydroepiandrosterone and androsterone in blood of mammals under physiological conditions and following administration of dehydroepiandrosterone. Acta Endocrinol. (1977) 85:126–33. 10.1530/acta.0.0850126 140574

[15] Brock BJ Waterman MR. Biochemical differences between rat and human cytochrome P450c17 support the different steroidogenic needs of these two species. Biochemistry. (1999) 38:1598–606. 10.1021/bi9821059 9931027

[16] Shet MS Fisher CW Tremblay Y Belanger A Conley AJ Mason JI Estabrook RW Comparison of the 17α-hydroxylase/C17,20-lyase activities of porcine, guinea pig and bovine P450c17 using purified recombinant fusion proteins containing P450c17 linked to NADPH-P450 reductase. Drug Metab Rev. (2007) 39:289–307. 10.1080/03602530701468391 17786622

[17] Turcu AF Rege J Auchus RJ Rainey WE. 11-Oxygenated androgens in health and disease. Nat Rev Endocrinol. (2020) 16:284–96. 10.1038/s41574-020-0336-x 32203405 PMC7881526

[18] Mueller JW Gilligan LC Idkowiak J Arlt W Foster PA. The regulation of steroid action by sulfation and desulfation. Endocr Rev. (2015) 36:526–63. 10.1210/er.2015-1036 26213785 PMC4591525

[19] Kaminska B Opalka M Ciereszko RE Dusza L. The involvement of prolactin in the regulation of adrenal cortex function in pigs. Domest Anim Endocrinol. (2000) 19:147–57. 10.1016/S0739-7240(00)00076-X 11064218

[20] Le Roy C Li JY Stocco DM Langlois D Saez JM. Regulation by adrenocorticotropin (ACTH), angiotensin II, transforming growth factor-β, and insulin-like growth factor I of bovine adrenal cell steroidogenic capacity and expression of ACTH receptor, steroidogenic acute regulatory protein, cytochrome P450. Endocrinology. (2000) 141:1599–607. 10.1210/endo.141.5.7457 10803567

[21] Woods AM Judd AM. Interleukin-4 increases cortisol release and decreases adrenal androgen release from bovine adrenal cells. Domest Anim Endocrinol. (2008) 34:372–82. 10.1016/j.domaniend.2007.10.004 18055157

[22] Woods AM McIlmoil CJ Rankin EN Packer AA Stevens JC Macievic JA. Leukemia inhibitory factor protein and receptors are expressed in the bovine adrenal cortex and increase cortisol and decrease adrenal androgen release. Domest Anim Endocrinol. (2008) 35:217–30. 10.1016/j.domaniend.2008.05.005 18638665

[23] McIlmoil S Call GB Barney M Strickland J Judd AM. Interleukin-6 inhibits adrenal androgen release from bovine adrenal zona reticularis cells by inhibiting the expression of steroidogenic proteins. Domest Anim Endocrinol. (2015) 53:108–23. 10.1016/j.domaniend.2015.05.006 26218834

[24] Marinelli L Trevisi E Da Dalt L Merlo M Bertoni G Gabai G. Dehydroepiandrosterone secretion in dairy cattle is episodic and unaffected by ACTH stimulation. J Endocrinol. (2007) 194:627–35. 10.1677/JOE-07-0226 17761902

[25] Jurkovich V Bakony M Laky E Ruff F Kézér FL Bende A. Cardiac vagal tone, plasma cortisol, and dehydroepiandrosterone response to an ACTH challenge in lame and nonlame dairy cows. Domest Anim Endocrinol. (2020) 71:106388. 10.1016/j.domaniend.2019.106388 31821929

[26] Frank LA Rohrbach BW Bailey EM West JR Oliver JW. Steroid hormone concentration profiles in healthy intact and neutered dogs before and after cosyntropin administration. Domest Anim Endocrinol. (2003) 24:43–57. 10.1016/S0739-7240(02)00204-7 12450624

[27] Skarlandtová H Bičiková M NeuŽil P Mlček M Hrachovina V Svoboda T. Are there any differences between stress hormone levels in non-stress conditions and in potentional stress overload (heart catheterisation) in sows? Physiol Res. (2014) 63:733–41. 25157655 10.33549/physiolres.932762

[28] Mapes S Corbin CJ Tarantal A Conley A. The primate adrenal zona reticularis is defined by expression of cytochrome b5, 17alpha-hydroxylase/17,20-lyase cytochrome P450 (P450c17) and NADPH-cytochrome P450 reductase (reductase) but not 3beta-hydroxysteroid dehydrogenase/delta5-4 isomerase (3beta-HSD). J Clin Endocrinol Metab. (1999) 84:3382–5. 10.1210/jcem.84.9.6105 10487714

[29] Rainey WE Nakamura Y. Regulation of the adrenal androgen biosynthesis. J Steroid Biochem Mol Biol. (2008) 108:281–6. 10.1016/j.jsbmb.2007.09.015 17945481 PMC2699571

[30] Parker CR. Dehydroepiandrosterone and dehydroepiandrosterone sulfate production in the human adrenal during development and aging. Steroids. (1999) 64:640–7. 10.1016/S0039-128X(99)00046-X 10503722

[31] Cloutier M Fleury A Courtemanche J Ducharme L Mason JI Lehoux JG. Characterization of the adrenal cytochrome P450C17 in the hamster, a small animal model for the study of adrenal dehydroepiandrosterone biosynthesis. DNA Cell Biol. (1997) 16:357–68. 10.1089/dna.1997.16.357 9115645

[32] Schiebinger RJ Albertson BD Barnes KM Cutler GB Loriaux DL. Developmental changes in rabbit and dog adrenal function: a possible homologue of adrenarche in the dog. Am J Physiol Endocrinol Metab. (1981) 3:694–9. 10.1152/ajpendo.1981.240.6.E694 6264794

[33] Hornsby PJ Aldern KA. Steroidogenic enzyme activities in cultured human definitive zone adrenocortical cells: comparison with bovine adrenocortical cells and resultant differences in adrenal androgen synthesis. J Clin Endocrinol Metab. (1984) 58:121–7. 10.1210/jcem-58-1-121 6315754

[34] Mongillo P Prana E Gabai G Bertotto D Marinelli L. Effect of age and sex on plasma cortisol and dehydroepiandrosterone concentrations in the dog (Canis familiaris). Res Vet Sci. (2014) 96:33–8. 10.1016/j.rvsc.2013.10.010 24269080

[35] Schiffer L Arlt W Storbeck KH. Intracrine androgen biosynthesis, metabolism and action revisited. Mol Cell Endocrinol. (2018) 465:4–26. 10.1016/j.mce.2017.08.016 28865807 PMC6565845

[36] Robic A Feve K Louveau I Riquet J Prunier A Exploration of steroidogenesis-related genes in testes, ovaries, adrenals, liver and adipose tissue in pigs. Anim Sci J. (2016) 87:1041–7. 10.1111/asj.12532 27436769

[37] Tan HS Raeside JI Developmental patterns of plasma dehydroepiandrosterone sulfate and testosterone in male pigs. Anim Reprod Sci. (1980) 3:73–81. 10.1016/0378-4320(80)90032-9

[38] Schuler G Dezhkam Y Bingsohn L Hoffmann B Failing K Galuska CE Free and sulfated steroids secretion in postpubertal boars (Sus scrofa domestica). Reproduction. (2014) 148:303–14. 10.1530/REP-14-0193 24961601

[39] Klymiuk MC Neunzig J Bernhardt R Sánchez-Guijo A Hartmann MF Wudy SA. Efficiency of the sulfate pathway in comparison to the Δ4- and Δ5-pathway of steroidogenesis in the porcine testis. J Steroid Biochem Mol Biol. (2018) 179:64–72. 10.1016/j.jsbmb.2017.10.017 29107177

[40] Kucera H Puschner B Conley A Berger T. Tissue steroid levels in response to reduced testicular estrogen synthesis in the male pig, Sus scrofa. PLoS ONE. (2019) 14:1–18. 10.1371/journal.pone.0215390 30986232 PMC6464225

[41] Holcenberg JS Rosen SW. Enzymatic sulfation of steroids by bovine tissues. Arch Biochem Biophys. (1965) 110:551–7. 10.1016/0003-9861(65)90449-2 4221028

[42] Lacoste D Dubè D Trudel C Bélanger A Labrie F. Normal gonadal functions and fertility after 23 months of treatment of prepubertal male and female dogs with the GnRh agonist [D-Trp6, des-Gly-NH210]GnRH ethylamide. J Androl. (1989) 10:456–65. 10.1002/j.1939-4640.1989.tb00140.x 2695507

[43] Wise TH Caton D Thatcher WW Lehrer AR Fields MJ. Androstenedione, dehydroepiandrosterone and testosterone in ovarian vein plasma and androstenedione in peripheral arterial plasma during the bovine oestrous cycle. J Reprod Fertil. (1982) 66:513–8. 10.1530/jrf.0.0660513 6217325

[44] Dieleman SJ Bevers MM Poortman J Van Tol HT. Steroid and pituitary hormone concentrations in the fluid of preovulatory bovine follicles relative to the peak of LH in the peripheral blood. J Reprod Fertil. (1983) 69:641–9. 10.1530/jrf.0.0690641 6226779

[45] Arlotto MD Michael MD Kilgore MW Simpson ER. 17α-Hydroxylase gene expression in the bovine ovary: mechanisms regulating expression differ from those in adrenal cells. J Steroid Biochem Mol Biol. (1996) 59:21–9. 10.1016/S0960-0760(96)00088-X 9009234

[46] Marinelli L Gabai G Simontacchi C Bono G. Effect of aging and reproductive condition on dehydroepiandrosterone plasma levels in the bitch. Vet Res Commun. (2007) 31:169–72. 10.1007/s11259-007-0024-5 17682867

[47] Tagliaferro AR Ronan AM. Physiological levels and action of dehydroepiandrosterone in Yucatan miniature swine. Am J Physiol Regul Integr Comp Physiol. (2001) 281:1–9. 10.1152/ajpregu.2001.281.1.R1 11404272

[48] Cooke GM. Steroidogenesis in interstitial cells and microsomal fraction of immature pig testes. J Reprod Fertil. (1991) 91:175–85. 10.1530/jrf.0.0910175 1847421

[49] Almeida J Conley AJ Mathewson L Ball BA. Expression of steroidogenic enzymes during equine testicular development. Reproduction. (2011) 141:841–8. 10.1530/REP-10-0499 21300693

[50] Bedrak E Samuels LT. Steroid biosynthesis by the equine testis. Endocrinology. (1969) 85:1186–95. 10.1210/endo-85-6-1186 4390633

[51] Hoffmann B Landeck A. Testicular endocrine function, seasonality and semen quality of the stallion. Anim Reprod Sci. (1999) 57:89–98. 10.1016/S0378-4320(99)00050-0 10565441

[52] Strauss JF Martinez F Kiriakidou M. Placental steroid hormone synthesis: unique features and unanswered questions. Biol Reprod. (1996) 54:303–11. 10.1095/biolreprod54.2.303 8788180

[53] Legacki EL Ball BA Corbin CJ Loux SC Scoggin KE Stanley SD. Equine fetal adrenal, gonadal and placental steroidogenesis. Reproduction. (2017) 154:445–54. 10.1530/REP-17-0239 28878092

[54] Legacki EL Scholtz EL Ball BA Stanley SD Berger T Conley AJ. The dynamic steroid landscape of equine pregnancy mapped by mass spectrometry. Reproduction. (2016) 151:421–30. 10.1530/REP-15-0547 26814209

[55] Satué K Marcilla M Medica P Ferlazzo A Fazio E. Testosterone, androstenedione and dehydroepiandrosterone concentrations in pregnant Spanish Purebred mare. Theriogenology. (2019) 123:62–7. 10.1016/j.theriogenology.2018.09.025 30292857

[56] Legacki EL Scholtz EL Ball BA Esteller-Vico A Stanley SD Conley AJ. Concentrations of sulphated estrone, estradiol and dehydroepiandrosterone measured by mass spectrometry in pregnant mares. Equine Vet J. (2019) 51:802–8. 10.1111/evj.13109 30891816

[57] Geisert RD Conley AJ Secretion and metabolism of steroids in subprimate mammals during pregnancy. In: Bazer FW, editor. Endocrinology of Pregnancy. Contemporary Endocrinology, Vol. 9. Totowa, NJ: Humana Press (1998). p. 291–318. 10.1007/978-1-4612-1804-3_10

[58] Gabai G Marinelli L Simontacchi C Bono GG. The increase in plasma C19Δ5 steroids in subcutaneous abdominal and jugular veins of dairy cattle during pregnancy is unrelated to estrogenic activity. Steroids. (2004) 69:121–7. 10.1016/j.steroids.2003.12.001 15013690

[59] Corpechot C Robel P Axelson M Sjövall J Baulieu EE. Characterization and measurement of dehydroepiandrosterone sulfate in rat brain. Proc Nat Acad Sci USA. (1981) 78:4704–7. 10.1073/pnas.78.8.4704 6458035 PMC320231

[60] Baulieu EE Robel P. Dehydroepiandrosterone (DHEA) and dehydroepiandrosterone sulfate (DHEAS) as neuroactive neurosteroids. Proc Nat Acad Sci USA. (1998) 95:4089–91. 10.1073/pnas.95.8.4089 9539693 PMC34265

[61] Wolf OT Kirschbaum C. Actions of dehydroepiandrosterone and its sulfate in the central nervous system: effects on cognition and emotion in animals and humans. Brain Res Rev. (1999) 30:264–88. 10.1016/S0165-0173(99)00021-1 10567728

[62] Soma KK Rendon NM Boonstra R Albers HE Demas GE. DHEA effects on brain and behavior: insights from comparative studies of aggression. J Steroid Biochem Mol Biol. (2015) 145:261–72. 10.1016/j.jsbmb.2014.05.011 24928552

[63] Giannini A Caretto M Genazzani AR Simoncini T. Optimizing quality of life through sex steroids by their effects on neurotransmitters. Climacteric. (2019) 22:55–9. 10.1080/13697137.2018.1543265 30570355

[64] Yilmaz C Karali K Fodelianaki G Gravanis A Chavakis T Charalampopoulos I. Neurosteroids as regulators of neuroinflammation. Fron Neuroendocrinol. (2019) 55:100788. 10.1016/j.yfrne.2019.100788 31513776

[65] Greaves RF Wudy SA Badoer E Zacharin M Hirst JJ Quinn T. A tale of two steroids: the importance of the androgens DHEA and DHEAS for early neurodevelopment. J Steroid Biochem Mol Biol. (2019) 188:77–85. 10.1016/j.jsbmb.2018.12.007 30557606

[66] Strac DS Konjevod M Perkovic MN Tudor L Erjavec GN Pivac N. Dehydroepiandrosterone (DHEA) and its sulphate (DHEAS) in Alzheimer's disease. Curr Alzheimer Res. (2020) 17:141–57. 10.2174/1567205017666200317092310 32183671

[67] Do Rego J-L Sujata A Jae YS Ludovic G David A Patrice B. Vasotocin and mesotocin stimulate the biosynthesis of neurosteroids in the frog brain. J Neurosci. (2006) 26:6749–60. 10.1523/JNEUROSCI.4469-05.2006 16793882 PMC6673836

[68] Newman AEM Soma KK. Aggressive interactions differentially modulate local and systemic levels of corticosterone and DHEA in a wild songbird. Horm Behav. (2011) 60:389–96. 10.1016/j.yhbeh.2011.07.007 21784076

[69] Diotel N Do Rego J-L Anglade I Vaillant C Pellegrini E Gueguen M-M. Activity and expression of steroidogenic enzymes in the brain of adult zebrafish. Eur J Neurosci. (2011) 34:45–56. 10.1111/j.1460-9568.2011.07731.x 21692878

[70] Caruso D Pesaresi M Abbiati F Calabrese D Giatti S Garcia-Segura LM. Comparison of plasma and cerebrospinal fluid levels of neuroactive steroids with their brain, spinal cord and peripheral nerve levels in male and female rats. Psychoneuroendocrinology. (2013) 38:2278–90. 10.1016/j.psyneuen.2013.04.016 23706961

[71] Kancheva R Hill M Novák Z Chrastina J Kancheva L Stárka L. Neuroactive steroids in periphery and cerebrospinal fluid. Neuroscience. (2011) 191:22–7. 10.1016/j.neuroscience.2011.05.054 21641969

[72] Rammouz G Lecanu L Papadopoulos V. Oxidative stress-mediated brain dehydroepiandrosterone (DHEA) formation in Alzheimer's disease diagnosis. Front Endocrinol. (2011) 2:69. 10.3389/fendo.2011.00069 22654823 PMC3356139

[73] Park I-H Han B-K Jo D-H. Distribution and characterization of neurosteroid sulfatase from the bovine brain. J Steriod Biochem Mol Biol. (1997) 62:315–20. 10.1016/S0960-0760(97)00042-3 9408085

[74] Yarim M Kabakci N. Neurosteroidogenesis in oligodendrocytes and Purkinje neurones of cerebellar cortex of dogs. J Vet Med C Anat Histol Embryol. (2004) 33:151–4. 10.1111/j.1439-0264.2004.00525.x 15144282

[75] Mongillo P Bernardini M Prana E Balducci F Gabai G Marinelli L Dehydroepiandrosterone and cortisol concentrations in the cerebrospinal fluid of dogs. Korean J Vet Res. (2017) 57:47–50. 10.14405/kjvr.2017.57.1.47

[76] Conley AJ Bernstein RM Nguyen AD. Adrenarche in nonhuman primates: the evidence for it and the need to redefine it. J Endocrinol. (2012) 214:121–31. 10.1530/JOE-11-0467 22378920

[77] Ogo A Haji M Ohashi M Nawata H. Decreased expression of cytochrome P450 17 alpha-hydroxylase mRNA in senescent bovine adrenal gland. Gerontology. (1991) 37:262–71. 10.1159/000213270 1660010

[78] Collomp K Baillot A Forget H Coquerel A Rieth N Vibarel-Rebot N. Altered diurnal pattern of steroid hormones in relation to various behaviors, external factors and pathologies: a review. Physiol Behav. (2016) 164:68–85. 10.1016/j.physbeh.2016.05.039 27235338

[79] Hucklebridge F Hussain T Evans P Clow A. The diurnal patterns of the adrenal steroids cortisol and dehydroepiandrosterone (DHEA) in relation to awakening. Psychoneuroendocrinology. (2005) 30:51–7. 10.1016/j.psyneuen.2004.04.007 15358442

[80] Rosenfeld RS Rosenberg BJ Fukushima DK Hellman L. 24-hour secretory pattern of dehydroisoandrosterone and dehydroisoandrosterone sulfate. J Clin Endocrinol Metab. (1975) 40:850–5. 10.1210/jcem-40-5-850 123927

[81] Lejeune-Lenain C Van Cauter E Désir D Beyloos M Franckson JRM. Control of circadian and episodic variations of adrenal androgens secretion in man. J Endocrinol Investig. (1987) 10:267–76. 10.1007/BF03348129 2957420

[82] Cook NJ Review: minimally invasive sampling media and the measurement of corticosteroids as biomarkers of stress in animals. Can J Anim Sci. (2012) 92:227–59. 10.4141/cjas2012-045

[83] Arvat E Di Vito L Lanfranco F Maccario M Baffoni C Rossetto R. Stimulatory effect of adrenocorticotropin on cortisol, aldosterone, and dehydroepiandrosterone secretion in normal humans: dose-response study. J Clin Endocrinol Metab. (2000) 85:3141–6. 10.1210/jcem.85.9.6784 10999799

[84] Pavlov EP Harman SM Chrousos GP Loriaux DL Blackman MR. Responses of plasma adrenocorticotropin, cortisol, and dehydroepiandrosterone to ovine corticotropin-releasing hormone in healthy aging men. J Clin Endocrinol Metab. (1986) 62:767–72. 10.1210/jcem-62-4-767 3005357

[85] Hung TT LeMaire WJ. The effects of corticotropin, opioid peptides and crude pituitary extract on the production of dehydroepiandrosterone and corticosterone by mature rat adrenal cells in tissue culture. J Steroid Biochem. (1988) 29:721–6. 10.1016/0022-4731(88)90174-4 2968481

[86] Torres JM Ortega E. DHEA, PREG and their sulphate derivatives on plasma and brain after CRH and ACTH administration. Neurochem Res. (2003) 28:1187–91. 10.1023/A:1024276328127 12834258

[87] Fenske M. Adrenal function in the mongolian gerbil (*Meriones unguiculatus*): influence of confinement stress upon glucocorticosteroid, progesterone, dehydroepiandrosterone, testosterone and androstenedione plasma levels, adrenal content and *in-vitro* secretion. Exp Clin Endocrinol Diabetes. (1986) 87:15–25. 10.1055/s-0029-1210517 3017731

[88] Bellanger S Battista MC Fink GD Baillargeon JP. Saturated fatty acid exposure induces androgen overproduction in bovine adrenal cells. Steroids. (2012) 77:347–53. 10.1016/j.steroids.2011.12.017 22245830 PMC3848974

[89] Fokidis HB. Sources of variation in plasma corticosterone and dehydroepiandrosterone in the male northern cardinal (*Cardinalis cardinalis*): I. Seasonal patterns and effects of stress and adrenocorticotropic hormone. Gen Comp Endocrinol. (2016) 235:192–200. 10.1016/j.ygcen.2016.05.024 27255363

[90] Higuchi K Nawata H Maki T Higashizima M Kato KI Ibayashi H. Prolactin has a direct effect on adrenal androgen secretion. J Clin Endocrinol Metab. (1984) 59:714–8. 10.1210/jcem-59-4-714 6090494

[91] Higuchi K Nawata H Kato K Ibayashi H. Ovine prolactin potentiates the action of adrenocorticotropic hormone on the secretion of dehydroepiandrosterone sulfate and dehydroepiandrosterone from cultured bovine adrenal cells. Horm Metab Res. (1985) 17:451–3. 10.1055/s-2007-1013574 2995221

[92] Judd AM Call GB Barney M McIlmoil CJ Balls AG Adams A. Possible function of IL-6 and TNF as intraadrenal factors in the regulation of adrenal steroid secretion. Ann N Y Acad Sci. (2006) 917:628–37. 10.1111/j.1749-6632.2000.tb05428.x 11268391

[93] Pepe GJ Albrecht ED Pepe GJ Albrecht ED. Prolactin stimulates adrenal androgen secretion in infant baboons. Endocrinology. (1985) 117:1968–73. 10.1210/endo-117-5-1968 2995004

[94] Kroboth PD Salek FS Pittenger AL Fabian TJ Frye RF. DHEA and DHEA-S : a review. J Clin Pharmacol. (1999) 39:327–48. 10.1177/00912709922007903 10197292

[95] Walker FR Pfingst K Carnevali L Sgoifo A Nalivaiko E. In the search for integrative biomarker of resilience to psychological stress. Neurosci Biobehav Rev. (2017) 74:310–20. 10.1016/j.neubiorev.2016.05.003 27179452

[96] Straub RH Lehle K Herfarth H Weber M Falk W Preuner J. Dehydroepiandrosterone in relation to other adrenal hormones during an acute inflammatory stressful disease state compared with chronic inflammatory disease: role of interleukin-6 and tumour necrosis factor. Eur J Endocrinol. (2002) 146:365–74. 10.1530/eje.0.1460365 11888843

[97] Lennartsson AK Kushnir MM Bergquist J Jonsdottir IH. DHEA and DHEA-S response to acute psychosocial stress in healthy men and women. Biol Psychol. (2012) 90:143–9. 10.1016/j.biopsycho.2012.03.003 22445967

[98] Lennartsson AK Theorell T Kushnir MM Bergquist J Jonsdottir IH. Perceived stress at work is associated with attenuated DHEA-S response during acute psychosocial stress. Psychoneuroendocrinology. (2013) 38:1650–7. 10.1016/j.psyneuen.2013.01.010 23428256

[99] Gabai G Amadori M Knight CH Werling D. The immune system is part of a whole-organism regulatory network. Res Vet Sci. (2018) 116:1–3. 10.1016/j.rvsc.2017.09.018 28958409

[100] Turnbull AV Rivier CL. Regulation of the hypothalamic-pituitary-adrenal axis by cytokines: actions and mechanisms of action. Physiol Rev. (1999) 79:1–71. 10.1152/physrev.1999.79.1.1 9922367

[101] Bornstein SR Rutkowski H Vrezas I. Cytokines and steroidogenesis. Mol Cell Endocrinol. (2004) 215:135–41. 10.1016/j.mce.2003.11.022 15026186

[102] Falomo ME Del Re B Rossi M Giaretta E Da Dalt L Gabai G. Relationship between postpartum uterine involution and biomarkers of inflammation and oxidative stress in clinically healthy mares (*Equus caballus*). Heliyon. (2020) 6:e03691. 10.1016/j.heliyon.2020.e03691 32258514 PMC7125350

[103] Valacchi G Virgili F Cervellati C Pecorelli A. OxInflammation: from subclinical condition to pathological biomarker. Front Physiol. (2018) 9:858. 10.3389/fphys.2018.00858 30038581 PMC6046448

[104] Colitti M Stefanon B Gabai G Gelain M Bonsembiante F. Oxidative stress and nutraceuticals in the modulation of the immune function: current knowledge in animals of veterinary interest. Antioxidants. (2019) 8:28. 10.3390/antiox8010028 30669304 PMC6356544

[105] Gundlach NH Feldmann M Gundelach Y Gil MA Siebert U Hoedemaker M. Dehydroepiandrosterone and cortisol/dehydroepiandrosterone ratios in dairy cattle with postpartum metritis. Res Vet Sci. (2017) 115:530–3. 10.1016/j.rvsc.2017.09.024 29055273

[106] Green MP Ledgard AM Beaumont SE Berg MC McNatty KP Peterson AJ. Long-term alteration of follicular steroid concentrations in relation to subclinical endometritis in postpartum dairy cows. J Anim Sci. (2011) 89:3551–60. 10.2527/jas.2011-3958 21666004

[107] Potterton SL Bell NJ Whay HR Berry EA Atkinson OCD Dean RS. A descriptive review of the peer and non-peer reviewed literature on the treatment and prevention of foot lameness in cattle published between 2000 and 2011. Vet J. (2012) 193:612–6. 10.1016/j.tvjl.2012.06.040 22951250

[108] Walker SL Smith RF Jones DN Routly JE Morris MJ Dobson H. The effect of a chronic stressor, lameness, on detailed sexual behaviour and hormonal profiles in milk and plasma of dairy cattle. Rep Domest Anim. (2010) 45:109–17. 10.1111/j.1439-0531.2008.01263.x 18992112

[109] Almeida PE Weber PSD Burton JL Zanella AJ. Depressed DHEA and increased sickness response behaviors in lame dairy cows with inflammatory foot lesions. Domest Anim Endocrinol. (2008) 34:89–99. 10.1016/j.domaniend.2006.11.006 17229542

[110] O'Driscoll K McCabe M Earley B Differences in leukocyte profile, gene expression, and metabolite status of dairy cows with or without sole ulcers. J Dairy Sci. (2015) 98:1685–95. 10.3168/jds.2014-8199 25557893

[111] O'Driscoll K McCabe M Earley B. Leukocyte profile, gene expression, acute phase response, and metabolite status of cows with sole hemorrhages. J Dairy Sci. (2017) 100:9382–91. 10.3168/jds.2017-13106 28843693

[112] Sprecher DJ Hostetler DE Kaneene J. A lameness scoring system that uses posture and gait to predict dairy cattle reproductive performance. Theriogenology. (1997) 47:1179–87. 10.1016/S0093-691X(97)00098-8 16728067

[113] Eckersall PD Bell R. Acute phase proteins: biomarkers of infection and inflammation in veterinary medicine. Vet J. (2010) 185:23–7. 10.1016/j.tvjl.2010.04.009 20621712

[114] Rondelli MCH Munhoz TD Catandi PB Freschi CR Palacios Junior RJG Machado RZ. Serum DHEA-S increases in dogs naturally infected with *Ehrlichia canis*. Res Vet Sci. (2015) 100:18–20. 10.1016/j.rvsc.2015.04.010 25956636

[115] Tejerizo G Doménech A Illera JC Silván G Gómez-Lucía E Altered plasma concentrations of sex hormones in cats infected by feline immunodeficiency virus or feline leukemia virus. Domest Anim Endocrinol. (2012) 42:113–20. 10.1016/j.domaniend.2011.11.001 22177694

[116] Clerici M Galli M Bosis S Gervasoni C Moroni M Norbiato G. Immunoendocrinologic abnormalities in human immunodeficiency virus infection. Ann N Y Acad Sci. (2000) 917:956–61. 10.1111/j.1749-6632.2000.tb05462.x 11268427

[117] Illera JC Pérez-Alenza MD Nieto A Jiménez MA Silvan G Dunner S Peña L. Steroids and receptors in canine mammary cancer. Steroids. (2006) 71:541–8. 10.1016/j.steroids.2005.11.007 16631217

[118] de Andrés PJ Illera JC Cáceres S Díez L Pérez-Alenza MD Peña L. Increased levels of interleukins 8 and 10 as findings of canine inflammatory mammary cancer. Vet Immunol Immunopathol. (2013) 152:245–51. 10.1016/j.vetimm.2012.12.010 23351639

[119] Aleman M Pickles KJ Conley AJ Stanley S Haggett E Toth B. Abnormal plasma neuroactive progestagen derivatives in ill, neonatal foals presented to the neonatal intensive care unit. Equine Vet J. (2013) 45:661–5. 10.1111/evj.12065 23600660

[120] Sporer KRB Xiao L Tempelman RJ Burton JL Earley B Crowe MA. Transportation stress alters the circulating steroid environment and neutrophil gene expression in beef bulls. Vet Immunol Immunopathol. (2008) 121:300–20. 10.1016/j.vetimm.2007.10.010 18061277

[121] Schurr MJ Fabian TC Croce MA Varnavas LE Proctor KG. Dehydroepiandrosterone, an endogenous immune modulator, after traumatic shock. Shock. (1997) 7:55–9. 10.1097/00024382-199701000-00007 8989837

[122] Fustini M Galeati G Gabai G Mammi LE Bucci D Baratta M. Overstocking dairy cows during the dry period affects dehydroepiandrosterone and cortisol secretion. J Dairy Sci. (2017) 100:620–8. 10.3168/jds.2016-11293 27837985

[123] Gross TS Williams WF. *In vitro*-steroid synthesis by the placenta of cows in late gestation and at parturition. J Reprod Fertil. (1988) 83:565–73. 10.1530/jrf.0.0830565 3411551

[124] Shenavai S Preissing S Hoffmann B Dilly M Pfarrer C Özalp GR. Investigations into the mechanisms controlling parturition in cattle. Reproduction. (2012) 144:279–92. 10.1530/REP-11-0471 22685253

[125] Lynch EM Earley B McGee M Doyle S. Characterisation of physiological and immunological responses in beef cows to abrupt weaning and subsequent housing. BMC Vet Res. (2010) 6: 10.1186/1746-6148-6-37 20646268 PMC2917423

[126] Labrie F. DHEA, important source of sex steroids in men and even more in women. Prog Brain Res. (2010) 182:97–148. 10.1016/S0079-6123(10)82004-7 20541662

[127] Labrie F. All sex steroids are made intracellularly in peripheral tissues by the mechanisms of intracrinology after menopause. J Steroid Biochem Mol Biol. (2015) 145:133–8. 10.1016/j.jsbmb.2014.06.001 24923731

[128] Nestler JE Advances in understanding the regulation and biologic actions of dehydroepiandrosterone. Curr Opin Endocrinol Diabetes Obes. (1996) 3:202–11. 10.1097/00060793-199606000-00003

[129] Traish AM Kang HP Saad F Guay AT Dehydroepiandrosterone (DHEA)-A precursor steroid or an active hormone in human physiology (CME). J Sex Med. (2011) 8:2960–82. 10.1111/j.1743-6109.2011.02523.x 22032408

[130] Klinge CM Clark BJ Prough RA. Dehydroepiandrosterone research: past, current, and future. Vitam Horm. (2018) 108:1–28. 10.1016/bs.vh.2018.02.002 30029723

[131] Prall SP Muehlenbein MP. DHEA modulates immune function: a review of evidence. Vitam Horm. (2018) 108:125–44. 10.1016/bs.vh.2018.01.023 30029724

[132] Molinari C Battaglia A Grossini E Mary DASG Vassanelli C Vacca G. The effect of dehydroepiandrosterone on coronary blood flow in prepubertal anaesthetized pigs. J Physiol. (2003) 549:937–44. 10.1113/jphysiol.2003.040170 12702737 PMC2342994

[133] Molinari C Battaglia A Grossini E Mary DASG Vassanelli C Vacca G. The effect of dehydroepiandrosterone on regional blood flow in prepubertal anaesthesized pigs. J Physiol. (2004) 557:307–19. 10.1113/jphysiol.2004.063354 15034120 PMC1665037

[134] Iruthayanathan M O'Leary B Paul G Dillon JS. Hydrogen peroxide signaling mediates DHEA-induced vascular endothelial cell proliferation. Steroids. (2011) 76:1483–90. 10.1016/j.steroids.2011.08.002 21864554

[135] Kurzman ID Panciera DL Miller JB. The effect of dehydroepiandrosterone combined with a low-fat diet in spontaneously obese dogs: a clinical trial. Obes Res. (1998) 6:20–8. 10.1002/j.1550-8528.1998.tb00310.x 9526966

[136] Van Der Stede Y Cox E Van Den broeck W Goddeeris BM. Enhanced induction of the IgA response in pigs by calcitriol after intramuscular immunization. Vaccine. (2001) 19:1870–8. 10.1016/S0264-410X(00)00440-0 11228356

[137] Burdick NC Dominguez JA Welsh TH Laurenz JC. Oral administration of dehydroepiandrosterone-sulfate (DHEAS) increases *in vitro* lymphocyte function and improves *in vivo* response of pigs to immunization against keyhole limpet hemocyanin (KLH) and ovalbumin. Int Immunopharmacol. (2009) 9:1342–6. 10.1016/j.intimp.2009.07.007 19646552

[138] Shen S Cooley DM Glickman LT Glickman N Waters DJ. Reduction in DNA damage in brain and peripheral blood lymphocytes of elderly dogs after treatment with dehydroepiandrosterone (DHEA). Mutat Res Fundam Mol Mech Mutagen. (2001) 480–481:153–62. 10.1016/S0027-5107(01)00179-8 11506809

[139] Maingat FG Polyak MJ Paul AM Vivithanaporn P Noorbakhsh F Ahboucha S. Neurosteroid-mediated regulation of brain innate immunity in HIV/AIDS: DHEA-S suppresses neurovirulence. FASEB J. (2013) 27:725–37. 10.1096/fj.12-215079 23150523

[140] Belvedere P Gabai G Valle LD Accorsi P Trivoletti M Colombo L. Occurrence of steroidogenic enzymes in the bovine mammary gland at different functional stages. J Steroid Biochem Mol Biol. (1996) 59:339–47. 10.1016/S0960-0760(96)00131-8 9010326

[141] Rijk JCW Bovee TFH Peijnenburg AACM Groot MJ Rietjens IMCM Nielen MWF. Bovine liver slices: a multifunctional *in vitro* model to study the prohormone dehydroepiandrosterone (DHEA). Toxicol In Vitro. (2012) 26:1014–21. 10.1016/j.tiv.2012.04.012 22640920

[142] Robinzon B Michael Miller KK Prough RA. Biosynthesis of [3H]7α-hydroxy-, 7β-hydroxy-, and 7-oxo-dehydroepiandrosterone using pig liver microsomal fractions. Anal Biochem. (2004) 333:128–35. 10.1016/j.ab.2004.06.003 15351289

[143] Lathe R Steroid and sterol 7-hydroxylation: ancient pathways. Steroids. (2002) 67:967–77. 10.1016/S0039-128X(02)00044-2 12398993

[144] Sollberger S Ehlert U How to use and interpret hormone ratios. Psychoneuroendocrinology. (2016) 63:385–97. 10.1016/j.psyneuen.2015.09.031 26521052

[145] Simontacchi C Perez De Altamirano T Marinelli L Angeletti R Gabai G Plasma steroid variations in bull calves repeatedly treated with testosterone, nortestosterone and oestradiol administered alone or in combination. Vet Res Commun. (2004) 28:467–77. 10.1023/B:VERC.0000040244.27933.f1 15509021

[146] Rijk JCW Peijnenburg AACM Hendriksen PJM Van Hende JM Groot MJ Nielen MWF. Feasibility of a liver transcriptomics approach to assess bovine treatment with the prohormone dehydroepiandrosterone (DHEA). BMC Vet Res. (2010) 6:44. 10.1186/1746-6148-6-44 20846423 PMC2949829

